# Effect of Guided Implant Drilling on Bone Temperature Changes During Implant Osteotomy: A Comprehensive Systematic Review

**DOI:** 10.7759/cureus.70216

**Published:** 2024-09-25

**Authors:** Vidushi Saxena, Pankaj Dhawan, Sapna Rani

**Affiliations:** 1 Department of Prosthodontics and Implantology, Manav Rachna Dental College, Faridabad, IND

**Keywords:** bone temperature, intraosseous heat generation, non-guided osteotomy, osteotomy, surgical guide

## Abstract

The preparation of the implant site and the quality of the bone are crucial factors for preliminary healing following implant surgery. Therefore, any thermal or mechanical damage to the bone must be minimized during osteotomy preparation. The use of guided implant drilling is now widely employed for the precise placement of implants. Although guided implants are accurate, they are said to generate more heat compared to non-guided osteotomy. This systematic review was conducted to evaluate the heat generation that occurs during osteotomy with both guided and non-guided drilling. A comprehensive search of dental literature in PubMed, EBSCOhost, and Google Scholar was performed for articles published from 2010 to 2024. The search strategy incorporated MeSH terms and Boolean operators. The initial search across all three databases yielded a total of 548 articles. Of these, 477 were discarded after reviewing the titles and abstracts, 26 were removed as duplicates, 15 studies were excluded due to unavailable full texts, and 19 were excluded for not meeting the study design criteria set for the systematic review. Eleven articles were ultimately selected for review and data extraction. After analyzing and collating the results from all the studies, it can be concluded that using surgical guides does cause significant heat generation in the bone at the osteotomy site. However, this rise in temperature generally remains below the threshold that could cause bone necrosis. Additionally, other factors, such as irrigation temperature, drill length, drill diameter, and drilling speed, also influence heat generation during osteotomy in guided drilling.

## Introduction and background

Rationale

Over the years, dental implant treatment has become a routine option for replacing lost teeth. Traditionally, dental implant surgeries involve making an incision in the mucosa over the crest of the alveolar ridge and elevating the mucoperiosteal flap to access the underlying bone. This is followed by implant placement, flap repositioning, and wound suturing [1]. With the growing need for increased precision and reduced surgery time, surgical guides have become popular due to their contribution to implant placement accuracy, time efficiency, and error minimization during surgery [2]. As the use of surgical guides has become widespread, there is a need to assess the effectiveness of various types of guides, particularly since some studies debate the use of surgical guides with irrigation cooling. Guided surgeries may limit the ability of irrigation liquid from the drill to directly reach the osteotomy site due to the presence of metal sleeves, which can significantly affect bone temperature. Bone temperature is critical during osteotomy, as exposure to temperatures of around 47°C for one minute is considered the threshold for causing irreversible osteonecrosis, making it essential to take precautions to avoid exposing the bone to thermal or mechanical damage [3,4].

Scope of the review

Primary healing after implant placement is a crucial process for achieving long-term success with dental implants. Both mechanical and thermal injuries during bone drilling can lead to bone tissue damage caused by heat. To avoid such damage, clinicians must be aware of the causes of heat generation and methods to prevent it. While guided surgeries have been widely adopted for their accuracy, their potential to generate heat requires a deep understanding of the contributing factors to mitigate temperature increases. This systematic review examines various studies assessing intraosseous heat generation during osteotomy preparation using surgical guides compared to non-guided osteotomies, along with other factors that must be considered to reduce potential increases in bone temperature when using surgical guides. Additional factors influencing heat generation include drill protocol (e.g., single or sequential drilling), drill diameter, drilling depth, the number of times the drill is used, rotational speed during osteotomy, and the temperature of the irrigation fluid.

Objective

Current literature on thermal fluctuations during implant osteotomy preparation focuses on drill material, drill diameter, drill shape, and intraosseous temperature changes associated with various implant preparation devices [5]. The objective of this review is to determine whether the use of surgical guides leads to irreversible bone damage due to potential heat generation compared to non-guided surgeries. An additional objective is to identify other factors that must be considered to minimize potential temperature increases when using surgical guides.

## Review

Materials and methods

**Figure 1:**
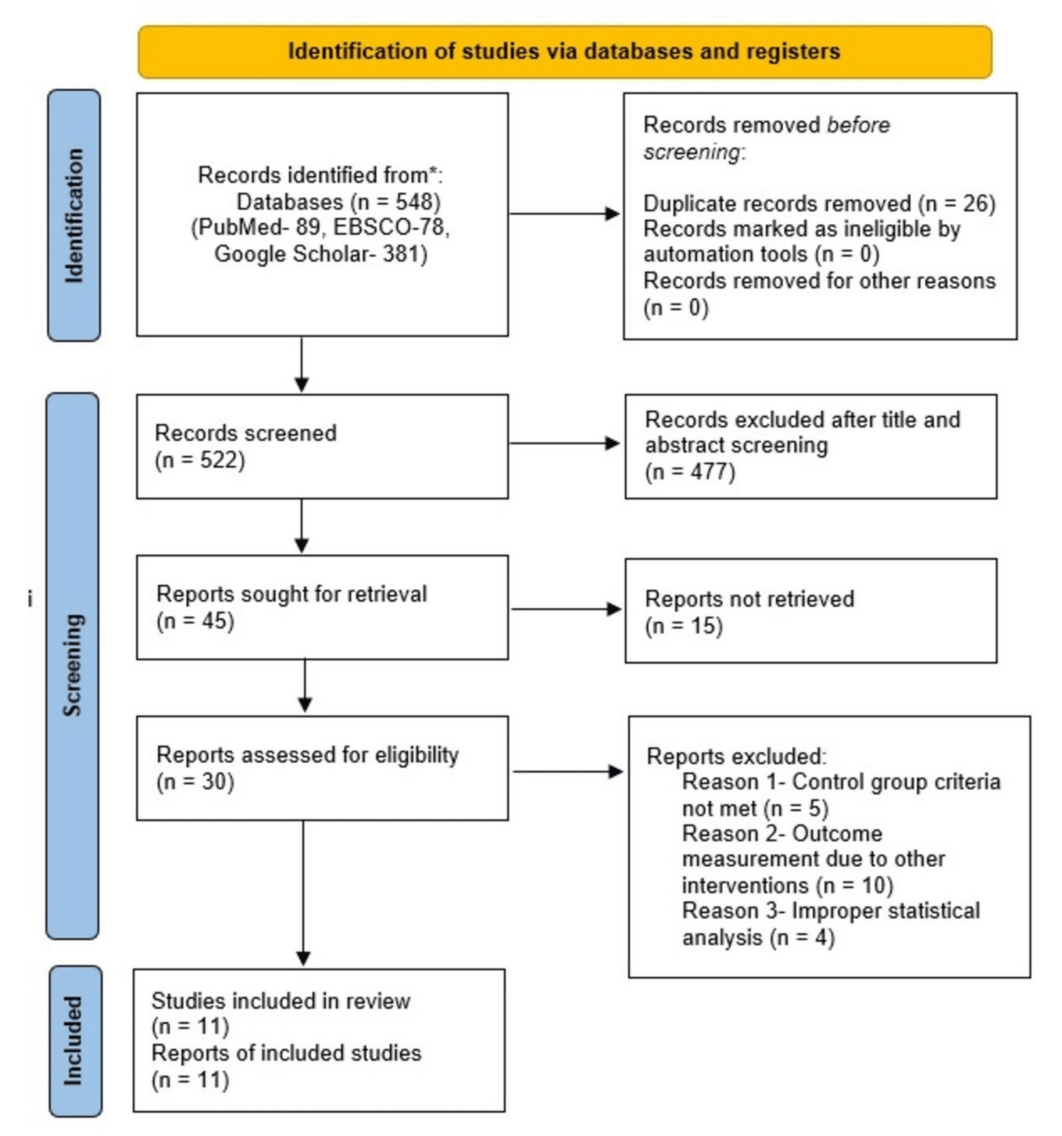
Flowchart of the study selection process based on the PRISMA guidelines PRISMA: Preferred Reporting Items for Systematic Reviews and Meta-Analysis

This systematic review followed the Preferred Reporting Items for Systematic Reviews and Meta-Analyses (PRISMA) guidelines (Figure 1).

The protocol was registered in the International Prospective Register of Systematic Reviews (PROSPERO; ID: CRD42024567119). The methods and protocol for the review were established and registered before conducting the actual review. A meta-analysis was planned to be conducted if deemed feasible. There were no deviations from the planned protocol as registered in PROSPERO.

The PICOS framework was used to identify the structure and components of this systematic review, along with a qualitative search strategy. The research question was as follows: “Does guided implant drilling affect temperature changes in bone during osteotomy preparation compared to non-guided implant drilling?”

The PICOS for the research question were as follows: P, bone models where implants will be placed; I, guided implant drilling procedures; C, non-guided drilling procedures; O, temperature changes in the bone; and S, types of studies (randomized control trials, non-randomized intervention studies, original research articles, and comparative studies). A search was conducted in PubMed, Google Scholar, and EBSCOhost using a predefined search strategy.

Search Strategy

A comprehensive search of available dental literature was conducted using PubMed, EBSCOhost, and Google Scholar for articles published from 2010 through 2024. A search strategy was developed using MeSH terms and Boolean operators. The search strategy was as follows: osteotomy AND surgical guide OR guided implant drill OR bone drilling AND temperature change OR bone temperature OR bone overheating OR osteonecrosis AND conventional osteotomy OR non-guided drilling AND dental implant.

Inclusion Criteria

The inclusion criteria were set to include full-text articles, randomized controlled trials, and non-randomized intervention studies. However, during the data search, we were unable to find randomized controlled trials, as most of the studies were in vitro and did not use any randomization of the comparative and control groups. Comparative experimental studies that examined conventional drilling versus all types of surgical guides, digital or conventional, limiting, partially limiting, or non-limiting, bone- or tissue-supported, were also included in the review.

Exclusion Criteria

Studies that focused on temperature changes due to factors such as drill design or material, bone density, drilling depth, and drill diameter, without mentioning surgical guides, were excluded. Additionally, studies with incomplete data, those not meeting the inclusion criteria, articles from fields other than dentistry, review articles, case reports, and case series were all excluded from the search.

Search Result

The initial search across all three databases yielded a total of 548 articles. Of these, 477 were discarded after reviewing the titles and abstracts, 26 were removed due to duplication, 15 were excluded because the full text was unavailable, and 19 were excluded for not meeting the PICOS criteria established for the systematic review. Ultimately, 11 articles were selected for review and data extraction.

Data Extraction

The results of the studies were tabulated under the following headings: author and year of publication, country, control group, any subgroups in the studies, type of bone model used for the study, type of drill used and drill speed, depth and diameter of the drill used, temperature of the irrigant, temperature measuring device, depth at which the temperature is measured, and the conclusion of the study (Tables 1-3).

**Table 1:** Characteristics of final studies included in the review based on the PRISMA flowchart for drilling protocols USA: United States of America, RPM: revolutions per minute (a measurement of rotational speed), mm: millimeter, PRISMA: Preferred Reporting Items for Systematic Reviews and Meta-Analyses

S. no.	Author and year of publication	Country	Bone type/bone model	Subgroups	Type of drill and RPM	Drill diameter and drill depth	Type of irrigation and temperature	Conclusion
1	Bulloch et al. (2012) [6]	Utah, USA	Bovine femoral bone	Single drill up to 3.5 mm, sequential drilling up to 4.2 mm	Straumann system drills at 2100 RPM	3.5 mm and 4.2 mm at 10 mm depth	Not specified	Mean temperature increases for guided was slightly (1°) higher than that of mean temperature increase for unguided
2	Jeong et al. (2013) [7]	South Korea	Resin mandible models with edentulous regions covered with a silicone lining mimicking the soft tissues and a wood block (D1 bone density) mimicking the hard tissues	Sequential drilling using the below-mentioned diameters: 2.2 mm, 2.8 mm, 3.2 mm, 3.8 mm, and 4.2 mm	A Dio drill system was used for implant site preparation at 1200 RPM speed	2 mm, 2.8 mm, 3.3 mm, 3.8 mm, and 4.3 mm diameter and 10 mm depth	Normal saline at room temperature	Drilling with external irrigation in an up-and-down pumping motion may not lead to a significant increase in bone temperature during a flapless procedure using surgical drill guides
3	Frösch et al. (2018) [3]	Switzerland	Polyurethane foam blocks	Single drill with 2.2 mm and sequential drilling with 2.2 mm, 2.8 mm, 3.5 mm, and 4.2 mm	Cylindrical pilot and twist drills 400-800 RPM	2.2 mm, 2.8 mm, 3.5 mm, and 4.2mm at 12 mm depth	Distilled water at 25°C	Use of surgical drill guides generates more heat than the conventional approach

**Table 2:** Characteristics of final studies included in the review based on the PRISMA flowchart for irrigation temperature RPM: revolutions per minute (a measurement of rotational speed), mm: millimeter, PRISMA: Preferred Reporting Items for Systematic Reviews and Meta-Analyses

S. no.	Author and year of publication	Country	Bone type/bone model	Subgroups	Type of drill and RPM	Drill diameter and drill depth	Type of irrigation and temperature	Conclusion
1	Marković et al. (2016) [8]	Serbia	Bovine ribs	Variable irrigation temperature of saline -25°C, -5°C	Pilot drill (SKYplanX, Bredent, Senden, Germany) at 600 RPM	2.35 mm drill and intraosseous depth of 13 mm	Saline at 25°C and cooled at 5°C	Guided implant site preparation generated a higher bone temperature than conventional drilling but not exceed a threshold for thermal bone necrosis
2	Boa et al. (2016) [9]	Hungary	Bovine ribs	Variable irrigation temperature of saline -10°C, -15°C, and -25°C	Type of drill is not specified, speed was 1200 RPM, type of drill was not specified, speed was 1200 RPM	Sequential drilling with 2.0, 2.5, 3.0-, and 3.5-mm burs	Irrigating saline at 10°C, 15°C, and 20°C temperature irrigating saline at 10°C, 15°C, and 20°C temperature	Use of saline as irrigating fluid at 10◦C meant that a significant reduction in peak temperature was achieved regardless of the site's preparation technique or the drill's diameter
3	Uçkun et al. (2023) [10]	Istanbul	Sheep iliac crest bones imitating D3 density	External irrigation at high speed (900 RPM), no irrigation at low speed (600 RPM), external irrigation at high speed with 24°C and 10°C	Type of drill was not specified, speed was 900 RPM or 300-600R PM	2.9 mm x 8.5 mm depth, 2.9 mm x 13 mm depth, 3.8 mm x 8.5 mm depth, 3.8 mm x 13 mm depth prepared as experimental groups	Irrigating saline at 24°C and 10°C	Surgical guide prevents the irrigation solution from reaching the area prepared for the implant, but intraosseous temperature can be kept within safe limits with external irrigation

**Table 3:** Characteristics of the final studies included in the review based on the PRISMA flowchart for drill properties RPM: revolutions per minute (a measurement of rotational speed), mm: millimeter, PU: polyurethane, DLC: diamond-like carbon coating, PRISMA: Preferred Reporting Items for Systematic Reviews and Meta-Analyses

S. no.	Author and year of publication	Country	Bone type/bone model	Subgroups	Type of drill and RPM	Drill diameter and drill depth	Type of irrigation and temperature	Conclusion
1	Migliorati et al. (2013) [5]	Italy	Pig ribs with a mean cortical thickness of 1.90 mm	Open flap with guided and conventional approach with two drill diameters, flapless with guided and conventional approach with two drill diameters	Twist drills (ACT twist drill, Biomet 3i) at 1200 RPM	2 mm and 3 mm drills at a depth of 12 mm	Room temperature saline (22.5°C) was used for irrigation	Site preparation with surgical stents generated higher bone temperature than conventional drilling. However, the heat generation did not reach temperature levels dangerous for the bone
2	dos Santos et al. (2014) [11]	Brazil	Tibias of rabbit	Number of times a drill was used 0 times, 10 times, 20 times, 30 times, and 40 times	Twist drills (Conexao sistemas de protese) at 1600 RPM	2 mm, 2.8 mm, 3 mm, and 3.15 mm at 4 mm depth	External irrigation with 0.9%, sodium chloride solution	Guided surgery technique generated a higher temperature than the classic drilling procedure. However, neither technique generated the critical bone temperature that bone can tolerate without necrosis
3	Barrak et al. (2018) [12]	Hungary	Bovine rib segments	Drilling speed of 1500 RPM and 2000 RPM, drill diameter of 2 mm, 2.5 mm, 3 mm, and 3.5 mm, irrigations temperature of 10°C, 15°C, and 20°C	Two fluted-drill bits with drill point angles of 90 degrees for 2 mm diameter and 120 degrees for 2.5, 3, and 3.5 mm diameters were used	Drill diameters of 2 mm, 2.5 mm, 3 mm, and 3.5 mm were used. Depth not specified	Normal saline at 10°C, 15°C, and 20°C was used	Both 1500 and 2000 RPM drilling can produce temperature elevations that exceed the necrotic threshold in a guided setting
4	Alhroob et al. (2021) [1]	Syria	Artificial bone block similar to human D2 bone (solid rigid polyurethane foam)	Different drill lengths of 6 mm, 8 mm, 10 mm, and 12 mm were used	Dentis bur at 1200 RPM	2.2 mm wide with depths of 6 mm, 8 mm, 10 mm, and 12 mm	85% irrigation used (temperature not specified)	Use of a surgical guide resulted in higher temperatures compared to a conventional method. However highest recorded temperature was below the bone necrosis threshold
5	Pupulin et al. (2024) [13]	Italy	Dental jaws made of commercial synthetic bone, consisting of rigid PU foams	Two types of guides: a resin jig that embeds metallic bushes into the guiding zones and a fully polymeric jig. External metallic adapters are used	For group A: stainless steel DLC coated to reduce friction with guide bushings at 400 RPM. For group B: drilling bits made of uncoated surgical-grade stainless steel at 700 RPM	Type A: 2.3 mm x 4 mm, 3.8 mm x 8.5 mm, and 3.8 mm x 15 mm. Type B: 2 mm x 4 mm, 2 mm x 12 mm, and 3.25 mm x 12 mm	Group A: no irrigation, Group B: irrigation with distilled water at 3°C below room temperature	Dental surgical guide systematically increases the tool temperature compared to a non-guided case. However, the magnitude of the introduced thermal effect is generally quite low, being quantified in an average of +4/5◦C, between the guided and the free surgery

Study Risk of Bias and Quality Assessment

Data from all individual studies included were independently reviewed by two reviewers. No articles were excluded upon reading the full-text literature. The Quality Assessment Tool For In Vitro Studies (QUIN tool) was used to analyze the risk of bias (Table 4) [14,15].

**Table 4:** Risk of bias assessment using QUIN tool Score: adequately specified: 2 points; inadequately specified: 1 point; not specified: 0 points; not applicable: NA. BIAS: low risk >70%; medium risk between 70% and 50%; high risk <50%. Bias evaluation: score x 100/2 divided by no. of criteria applicable. NA: not applicable, QUIN tool: Quality Assessment Tool For In Vitro Studies

S. no.	Article	Clearly stated aims and objectives	Detailed explanation of sample size calculation	Detailed explanation of the sampling technique	Details of the comparison group	Detailed methodology	Operator details	Randomization	Method of measurement of outcome	Outcome assessor details	Blinding	Statistical analysis	Presentation of results	Score	Bias evaluation
1.	Bulloch et al. (2012) [6]	2	0	0	2	2	1	1	2	0	NA	1	1	12	54.5 (medium risk)
2	Jeong et al. (2013) [7]	2	0	0	2	2	2	0	2	0	NA	1	1	12	54.5 (medium risk)
3	Migliorati et al (2013) [5]	2	0	0	2	2	2	0	2	1	NA	2	2	15	68.1 (medium risk)
4	dos Santos et al. (2014) [11]	2	0	0	2	2	1	0	2	1	NA	2	2	14	63.6 (medium risk)
5	Marković et al. (2016) [8]	2	0	2	2	2	2	2	2	1	NA	2	2	19	86.3 (low risk)
6	Boa et al. (2016) [9]	2	0	0	2	2	2	0	2	0	NA	2	2	14	63.6 (medium risk)
7	Barrak et al. (2018) [12]	2	0	0	2	2	2	0	2	0	NA	2	2	14	63.6 (medium risk)
8	Frösch et al. (2018) [3]	2	1	1	2	2	0	NA	2	1	NA	2	2	15	75 (low risk)
9	Alhroob et al. (2021) [1]	2	0	0	2	1	2	NA	2	0	NA	2	2	13	65 (medium risk)
10	Uçkun et al. (2023) [10]	2	0	0	2	1	1	0	2	0	NA	2	2	12	54.5 (medium risk)
11	Pupulin et al. (2024) [13]	2	0	0	2	2	2	NA	2	1	NA	2	2	15	75 (low risk)

The component of blinding in the QUIN tool was not applicable to our studies, as blinding the operator for osteotomy with guided and non-guided techniques was not possible. Additionally, the randomization component was not applicable to a few studies that used standardized artificial bone models for both groups. The risk of bias was calculated to be medium for eight studies, while three studies had a low risk of bias.

Meta-analysis

After reviewing all the articles, it was found that although the studies compared guided and non-guided surgical techniques for osteotomies, each study included additional varied parameters for comparison. Due to the presence of numerous subgroups and the heterogeneity of results across various studies, conducting a meta-analysis was not possible.

Results

Most studies included in this review concluded that guided implant surgeries generate some amount of heat at the time of osteotomy. A few of the studies compared the single and sequential drill protocols in their research, along with guided and non-guided surgeries. Bulloch et al. (2012) [6] compared guided osteotomies using single and sequential drill protocols. Their results were not significant in any comparative groups, except for guided drilling with a 3.5 mm diameter (sequential) compared to a cannulated drill, which yielded a p-value of 0.046. Jeong et al. (2013) [7] compared guided and non-guided drilling using the single drill protocol at 3 mm and 6 mm depths. The p-values between the two groups at 3 mm depth were 0.839, and at 6 mm depth, it was 0.142, both of which were non-significant. Frosch et al. (2018) [3] comparing a 2.2 mm single drill to sequential drilling showed a significant difference in guided osteotomy compared to the conventional approach, except for the single 2.2 mm drill. For the 4.2 mm drill, the differences were significant for both single and sequential drills using the guide and conventional approach (p=0.001 and p<0.001). Additionally, for conventional and guided drilling, the sequential as well as single drill protocols had statistically significant differences (p=0.001 and p=0.049).

Studies comparing different irrigation temperatures concluded that irrigation temperature had a significant effect on bone temperature. Marković et al. (2016) [8] compared guided osteotomy using saline at 5°C and 25°C. Their results indicated that surgical guides caused significant changes in bone at the entrance of the osteotomy. Additionally, the effect of temperature on saline at all levels of osteotomy was found to be significant (p<0.001). A rise in the temperature of the guide was significantly higher (p<0.001) with 25°C saline. Boa et al. (2016) [9] compared intraosseous temperature during guided and non-guided drilling using different irrigant temperatures (10°C, 15°C, and 20°C). According to their results, when freehand drilling with irrigation at 10°C was compared to guided drilling with irrigation at 15°C, there was a significant difference at 2.5 mm (p=0.005) and 3.0 mm (p=0.004), implicating the efficacy of irrigation at a lower temperature. When the guided group with an irrigation temperature of 10°C was compared to the freehand group with 25°C, there was a significant reduction in temperature values at all diameters except for 3.5 mm (p=0.431 for 2.0 mm, p=0.000 for 2.5 and 3.0 mm, while p=0.055 for 3.5 mm). Uçkun et al. (2023) [10] compared changes in temperature at the osteotomy site between traditional and guided methods. Group A used a guide and the conventional method with external cooling at high speed; Group B was the same as Group A but at low speed with no cooling; and Group C used guided and traditional methods at high speed with irrigation at 24°C and 10°C. There was a significant increase in temperature as the diameter increased for all groups. The p-value was found to be 0.001 for all groups. The changes in temperature increase between groups A and C, as well as between groups B and C, were statistically significant (p=0.001).

Migliorati et al. (2013) [5] compared open flap freehand surgery (OSS) and freehand flapless surgery to open flap guided surgery (OGS) and flapless guided surgical technique (FGS). The maximum temperature rise was found in the OGS group (median, 2.55°C), but there was no significant difference between the OGS and FGS groups (median, 2.50°C). Notable differences were observed when comparing the OGS with both the FSS and OSS groups, as well as when comparing the FGS group with both the FSS and OSS groups (p<.001). Dos Santos et al. (2014) [11] conducted a study comparing guided and traditional drilling, with subgroups divided based on the number of times a particular drill was used (G0, G1, G2, G3, and G4, denoting use 0 times, 10 times, 20 times, 30 times, and 40 times, respectively). The results indicated a significant difference in thermal oscillation between the conventional and guided groups, with the conventional group exhibiting lower temperatures. Regardless of the type of drill used, bone heating was found to be three times higher in the guided group. Alhroob et al. (2021) [1] compared heat generation for the two surgical techniques at different drill lengths. They found significant differences in heat generation between the conventional group (41.07°C) and the group using a surgical guide (42.97°C) (p<0.05). They also concluded that drill length was associated with changes in temperature, with more heat generated from longer drills (p<0.05). Barrak et al. (2019) [12] compared freehand and guided techniques along with various other parameters, including drilling speed, drill diameter, and irrigation fluid temperature. The study concluded that guided drilling with 20°C irrigation fluid at a speed of 1500 RPM yielded temperatures exceeding the threshold for necrosis (47°C) when performed with 3.0- and 3.5-mm-diameter bits. In contrast, 10°C irrigation fluid nullified significant statistical differences between the guided and freehand groups. Pupulin et al. (2024) [13] examined the evolution of temperature while drilling using two different methods for 3D-printed surgical guides: one employing an internal metal bushing system and the other utilizing external metal reducers. There was an average temperature rise of 2.4°C and 4.8°C of the drill shanks; however, the tips exhibited an increase of about 17°C and 24°C during freehand surgery and surgery using guides, respectively. Most studies used K-type thermocouples to measure temperature, whereas Marković et al. (2016) [8] employed a thermographic camera to measure temperature within the bone.

Discussion

Guided and Non-guided Osteotomy

Several studies have suggested that guided implant surgeries lead to higher bone temperatures because the irrigation liquid is unable to reach the point of osteotomy. Frösch et al. (2018) concluded that using surgical guides generated higher temperatures than the non-guided traditional approach. They suggested that the design of the implant drill may impact the development of heat and that intermittent drilling protocols and single drilling procedures could potentially reduce heat generation during guided surgeries [3]. Findings in the study by Alhroob et al. (2021) also supported these statements, as they concluded that a surgical guide could impede irrigation and thus lead to increased heat generation [1]. Drilling through a guide with a cooling channel has been shown to reduce temperature rise by a factor of 1.9, according to Liu et al. [16]. Marković et al. (2016) conducted a study that concluded that higher heat generation occurred during osteotomy with a guided surgical approach compared to conventional drilling. However, none of the surgical techniques created critical temperature values that could lead to bone necrosis [8]. According to their study, the drill guide's effect was significant only at the superficial level of the bony walls of osteotomy sites. Bulloch et al. (2012) mentioned other limitations of surgical splints, aside from heat generation, stating that they generally obstruct the view of the surgical field. Maintaining stability can be difficult, and the need for complex procedures like flap elevation or graft placement renders the surgical guides inaccurate and ineffective. Additionally, the tissue punch used in a few guided surgeries removes vital attached gingiva, which compromises the final result [6]. Therefore, it is up to the operator to weigh the benefits of surgical guides in particular cases against their challenges, including heat generation. Freehand osteotomies come with their own challenges, including errors by the operator and misalignment. Waltenberger et al. (2021) conducted similar studies in which they observed a temperature rise of 8.5°C when using a surgical guide [17]. Misir et al. (2009) also evaluated heat generation in bone with and without the use of a surgical guide and concluded that the preparation of osteotomy with a surgical drill guide creates more heat compared to conventional osteotomy preparation, regardless of the type of irrigation [18].

Effect of Drilling Protocol and Template Design

Waltenberger et al. (2021) mentioned that templates that are fully guided lead to a lesser quantity of irrigant reaching the osteotomy point [17]. There is an influence of template design on the total volume of irrigation liquid; hence, they suggested a pumping motion during drilling to avoid maximum temperature rise. In their study, they also observed that an “occlusal splint designed” drilling template with a lateral opening could lower the average temperature rise by 1.47, while the mean flow rate of irrigant could be increased by a factor of 1.4. Barrak et al. (2018) concluded that metal sleeves within guides are a major contributing factor to heat generation during guided osteotomy [19]. However, this effect may be reduced by limiting the number of osteotomies prepared with the same drill and by reducing the drilling speed. Bulloch et al. (2012) concluded that sequential drilling, whether or not using a surgical guide, led to heat generation, and they recommended the use of a cannulated single drill technique that does not lead to increased bone temperature [6]. Frösch et al. (2019) also suggested that higher temperatures were observed in sequential drilling compared to the single drill protocol [3]. Calvo-Guirado et al. (2014) tested a new hybrid protocol (simplified plus biologic drilling) for the preparation of implant sites without irrigation, which caused a temperature rise equivalent to that of the conventional incremental protocol [20].

Effect of Irrigation on Bone Temperature

Most of the studies concluded that cooling the irrigation fluid to lower temperatures reduces the amount of heat generated during osteotomy preparation. Boa et al. (2016) concluded that using pre-cooled irrigation fluid at 10°C is better than room temperature fluid in terms of temperature control [9]. Barrak et al. (2019) found in their study that drilling with guides at 20°C irrigation led to significantly higher temperature rise values compared to the conventional groups across all diameters. In contrast, with 15°C irrigation, the mean temperature elevations exceeded the limit for 3.0 mm and 3.5 mm diameter drills in the guided condition, suggesting the use of 10°C irrigation for higher diameter drills in guided conditions [12]. Uçkun et al. (2023) found that the temperature rise recorded in the group using a saline irrigation solution cooled to 10°C was notably less compared to irrigation with saline at 24°C [10]. Another study concluded that it is essential to use a cooling liquid to reduce the rise in temperature in bone, although the amount of liquid reaching the osteotomy site has a limited influence. The vital aspect is the continuous wetting of the drill to provide sufficient cooling, which can also be achieved using external irrigation [17].

Other Drill-Related Factors

Barrak et al. (2019) also studied the effect of drill speed on guided and freehand surgeries and concluded that both 1500 and 2000 RPM drilling can produce temperature elevations exceeding the necrotic threshold in a guided setting, with a speed of 2000 RPM potentially leading to harmful temperatures even with the freehand technique [12]. A study conducted in 2018 by the same authors concluded that at a speed of 800 RPM, the bone temperature never reaches the threshold for necrosis under any of the tested conditions in a total of 210 osteotomies they performed, even with the use of surgical guides [19]. Dos Santos et al. (2014) concluded that guided surgeries generated more heat compared to conventional procedures, and this temperature increase was directly proportional to the number of times a drill was used. Additionally, the surface roughness of the drill was directly proportional to the number of uses for either technique, but the guided technique led to increased surface roughness of the drill [11]. Ercoli et al. (2004) analyzed the impact of drill wear on cutting efficiency and correlated this factor to heat generation in bone. The conclusion was that the durability and cutting efficiency of drills are directly related to the drill's material and design [21].

Limitations of the Study

Most of the articles reviewed stated that the amount of heat generated and transferred by the implant drills to the bone during osteotomy depends on various factors, such as the speed of rotation, the cutting edge of the drill, drilling protocols (single or sequential), drilling depth, the design and shape of the drill, and the use of internal or external irrigation. The primary limitation of most studies is that none could be performed in vivo; instead, they used either animal bone or synthetic bone models. Moreover, a limitation of our review was that, due to stringent inclusion criteria, some studies comparing other factors were excluded and thus could not be reviewed in detail. Another limitation is that a meta-analysis could not be performed because, in most studies, p-values were measured for various cross-groups formed due to multiple comparative parameters.

## Conclusions

This systematic review includes studies that have compared heat generation during guided osteotomies to that during non-guided osteotomies. After analyzing and collating the results from all the studies, it can be concluded that the use of surgical guides does cause significant heat generation in the bone at the site of osteotomy; however, in general, this rise in temperature remains below the critical threshold that may lead to bone necrosis. Additionally, other factors such as irrigation temperature, drill length, drill diameter, and drilling speed play a role in heat generation during osteotomy with guided drilling. An increase in drill diameter and drill depth is expected to result in a temperature rise. This temperature rise can be controlled by using cooled irrigation liquid. In the case of fully limiting surgical guides with metal sleeves, a slow speed (400-800 RPM) along with refrigerated irrigation liquid is a viable option to reduce bone temperatures.
